# Introducing JMIR AI

**DOI:** 10.2196/42046

**Published:** 2022-09-20

**Authors:** Khaled El Emam, Bradley Malin

**Affiliations:** 1 School of Epidemiology and Public Health University of Ottawa Ottawa, ON Canada; 2 Department of Biomedical Informatics Vanderbilt University Nashville, TN United States

**Keywords:** artificial intelligence, AI, machine learning, methodology

## Abstract

JMIR AI is a new journal with a focus on publishing applied artificial intelligence and machine learning research. This editorial provides an overview of the primary objectives, the focus areas of the journal, and the types of articles that are within scope.

The past decade has witnessed rapid growth in the development of artificial intelligence and machine learning (AI/ML) methods for biomedical research and clinical applications. At the same time, it has become clear that the translation of such methods into practice has met numerous challenges. This is perhaps best exemplified by the status of AI/ML work during the COVID-19 pandemic. The pandemic was one moment in time where powerful AI/ML-driven diagnostic and prognostic tools could have accelerated our understanding and development of effective treatments. With some notable exceptions [1], and despite many publications, the impact of AI/ML in practice has been limited [2,3]. The reasons are varied [4-6], such as a lack of representation in the populations to whom the data corresponds and poor quality in the data available, leading to a lack of generalizable methodologies and models and a lingering lack of trust in automated decision-making. In this respect, our main motivation for JMIR AI is to publish articles that focus on the practical issues involved in developing useful AI/ML solutions and implementing them in biomedical research and clinical settings.

At the same time, we are seeing the introduction of policies and statutes in disparate jurisdictions to regulate AI/ML systems [7,8]. These policies and statutes are being developed in anticipation of an AI-laden future. Yet, as with all policy-making, such activities are likely to impact data access, the definition of fit-for-use data, algorithmic explainability and transparency, patient access to data and decision justifications, and the need for continuous evaluation of models in clinical settings, to name a few.

JMIR AI aims to become a venue for identifying, discussing, and addressing such practical challenges, with a particular emphasis on applications. The journal will strive to publish technical articles, as well as those tackling societal aspects, including ethical, legal, policy, and regulatory issues. This will be accomplished through a mix of research, perspectives, tutorials, and articles describing benchmark data sets. By leveraging JMIR Publications’ publishing processes and tools, we also expect to have a rigorous and rapid open access review and publication process.

To realize this vision, we are assembling a multidisciplinary editorial board covering a wide array of topics from academia and industry, as well as ensuring broad domain and regional representation. The founding members of the editorial board cover many years working in academic medical centers and with spin-off health technology companies, as well as working with and within the pharmaceutical and medical device industries. Given the continued rapid advancement of this multidisciplinary field, we intend to continue expanding the editorial board to cover relevant areas as they arise.

We also intend to use the journal as a platform to enable and facilitate code and data sharing. This will be achieved by providing authors with additional tools that address the many technical and regulatory obstacles to broader community sharing.

## Abbreviations

AI/ML: artificial intelligence and machine learning
